# Conference Report: MANUFACTURING TECHNOLOGY CONFERENCE: TOWARD A COMMON AGENDA Gaithersburg, MD April 18–20, 1995

**DOI:** 10.6028/jres.101.011

**Published:** 1996

**Authors:** David C. Stieren

**Affiliations:** National Institute of Standards and Technology, Gaithersburg, MD 20899-0001

## 1. Introduction

The second annual “Manufacturing Technology Conference: Toward a Common Agenda,” was held at the National Institute of Standards and Technology in Gaithersburg, Maryland, on April 18–20, 1995. The Conference helped define and refine the major elements of a national manufacturing agenda. Such an agenda involves consensus among government, industry, academia, and workforce organizations.

This year’s Conference built upon the progress made toward a common manufacturing agenda at last year’s priority-setting conference, which was attended by more than 600 participants. Attendance this year was approximately 250; a list of attendees is in the Conference Proceedings, NIST Special Publication 886 [1]. The Honorable Ronald Brown, Secretary of Commerce, delivered the Conference keynote address.

The Conference was planned by individuals from more than 30 government and industrial organizations. It was co-sponsored by the NIST Manufacturing Engineering Laboratory (MEL), the Conference host organization; the U.S. Department of Agriculture; the U.S. Department of Energy (DOE); the U.S. Navy, through the Navy’s Manufacturing Science and Technology Program; and Martin Marietta Energy Systems, through the Oak Ridge Centers for Manufacturing Technology.

## 2. Conference Program

The Conference focused primarily on a series of white papers that address critical manufacturing infrastructure issues. These white papers were prepared by the Committee of Civilian Industrial Technology/Manufacturing Infrastructure (CCIT/MI) Subcommittee, which is under the President’s National Science and Technology Council (NSTC). The following are the five “areas of emphasis,” along with a sixth area under consideration for inclusion, in the manufacturing infrastructure framework identified by the CCIT/MI:

- Advanced Manufacturing Systems,
- Engineering Tools for Design and Manufacturing,
- Manufacturing Processes and Equipment,
- Manufacturing Training and Education,
- Manufacturing Deployment, and
- Business Practices.

The Conference began with an overview of the work of the CCIT, followed by overviews of the Subcommittees for Manufacturing Infrastructure and Advanced Materials Processing. The overviews were presented by executives from the CCIT and its subcommittees. Following these presentation, executives from the following major industry sectors provided perspectives of the activities of the federal government relating to manufacturing, and how they impact industry:

- Aerospace, by James Sinnett, V.P. and General Manager, McDonnell Douglas Corporation, New Aircraft and Missile Products;
- Electronics, by Mauro Walker, Senior Vice President and Director of Manufacturing, Motorola, Inc.;
- Automotive, by Frank J. Ewasyshyn, Vice President, Chrysler Corporation;
- Heavy equipment, by Richard Thompson, Group President, Caterpillar, Inc.;
- Food, by Al Clausi, Past-President, Institute of Food Technologies;
- Apparel/textile, by Craig Long, Director of Quality, Miliken and Company; and
- Chemical, by James Schoonover, Director of Operations, DuPont Company.

The second day of the Conference shifted into a series of working concurrent panel sessions. These panel sessions provided participants an opportunity to evaluate the manufacturing framework proposed by the CCIT/MI. Each panel was a working group that focused on one of the six areas of emphasis in the manufacturing framework. Chaired by one representative from industry and one from government, each panel began with a context-setting presentation by an industry speaker. Panel members were charged with the tasks of defining appropriate public- and private-sector roles in the area of emphasis and recommending areas or activities for public/private collaboration. Feedback reports that summarized each panel’s discussion and conclusions were presented to a plenary session of the Conference later in the afternoon of the second day.

Prior to that plenary session, and while panel session reports were being prepared, representatives from NIST, the DOE, the Department of Defense (DOD), and the U.S. Secretariat of the international Intelligent Manufacturing Systems program provided overviews of ongoing and planned manufacturing-related activities.

The third day of the Conference focused on several specific federal manufacturing and technology programs. These included the NIST Advanced Technology Program (ATP) and Manufacturing Extension Partnership (MEP) Program; the DOD Manufacturing Science and Technology Program; the DOE Technologies Enabling Agile Manufacturing (TEAM) Program; the joint Technology Reinvestment Project (TRP); the international partnership for Intelligent Manufacturing Systems; and manufacturing programs at the National Science Foundation (NSF). The Conference concluded with tours of several NIST manufacturing facilities.

## 3. Conference Results

In addition to providing a national forum to exchange information about manufacturing technology among public and private organizations, the Conference had several working objectives. The working objectives were the definition and refinement of the NSTC manufacturing infrastructure areas of emphasis. The ultimate goal is continued progress toward a consensus manufacturing agenda for government, industry, and academia.

To this end, the Conference assembled working group panels according to participants’ interests to discuss the NSTC white papers and critical issues in each of the CCIT/MI areas of emphasis, as well as Business Practices. The following sections summarize the reports from each of the panels.

### 3.1 Advanced Manufacturing Systems

The Advanced Manufacturing Systems panel was co-chaired by a representative from NIST’s MEL and a representative of Westinghouse Electric Corporation. This area of emphasis deals with flexible and responsive manufacturing systems and enterprises that are needed to react to rapidly changing customer needs and market requirements.

Advanced manufacturing systems approach the process of incorporating new technologies and innovative business practices into enterprises by treating manufacturing systems and manufacturing enterprises as interdependent collections of the following:

- information systems, both technical and business;
- business practices and metrics;
- physical processes and equipment; and
- people.

The perspective is that in the manufacturing systems and enterprises of today and tomorrow cost-competitive products with high quality, performance, and functionality are manufactured in enterprises that leverage people, business, and technology resources. These enterprises are customer-focused and business-centered in such a way that technology supports what people direct. The enterprises must be reconfigurable, adaptive, and flexible. They must be based upon information and knowledge to facilitate global interoperability throughout the manufacturing cycle. They must also be modular in order to support systems that consist of distributed and autonomous units, with the various enterprise elements operating synergistically to achieve the goals of the enterprise.

Achieving a common agenda for advanced manufacturing systems requires the establishment of a government-industry manufacturing infrastructure investment strategy. This strategy should include innovative manufacturing systems concepts that are validated in technology demonstrations, and that are based upon enabling science and technologies. Examples of enabling science and technologies include the following:

- the national information infrastructure;
- national integration frameworks and standards, such as common architectures;
- integration tools to specify, design, evaluate, implement, and monitor the performance of manufacturing systems;
- analysis capabilities available through modeling and simulation; and
- intelligent controls and sensors.

Among the conclusions of the panel were the following.

Technology demonstrations should show how enabling sciences and technologies can be applied to business practices and services to enhance business cultures for the benefit of the enterprise.

The investment strategy for the government-industry manufacturing infrastructure should include international cooperation, and it should be based upon a common vision that is shared by the public and private sectors.

A key to the Advanced Manufacturing Systems area of emphasis is that business practices are an essential part of this nation’s manufacturing infrastructure. Poor U.S. business practices impede U.S. industry’s global competitiveness. The United States needs research and development (R&D) investment in business practices for next generation manufacturing enterprises just as much as it needs systems and technology R&D.

In the development of the nation’s manufacturing infrastructure, government and industry have distinct roles. The government should primarily facilitate interactions between stakeholders, and it should manage and improve regulatory influences. The government should provide funding for the creation of the infrastructure. The government should be a repository for global knowledge, and it should provide appropriate standards and standards methodologies.

Industry should primarily focus on business core competencies and the specifics of product applications and technologies.

### 3.2 Engineering Tools for Design and Manufacturing

The Engineering Tools for Design and Manufacturing panel was co-chaired by a representative of NIST’s MEL and a representative of General Electric. The panel’s area of emphasis was focused on engineering techniques for rapid and simultaneous development of new products, processes, and production systems. These techniques, or tools, are critical elements in reducing product development times, lowering manufacturing costs, eliminating inefficiencies, increasing product quality, and minimizing environmental impact. Examples of such tools are rapid prototyping, simulation, and modeling.

The panel discussed the NSTC white paper and identified the constraints within industry on the use of tools and data, such as supply chain interactions.

Tools must be validated within industry on industrial applications as part of their development process. A critical element in commercializing engineering tools for design and manufacturing is their affordability. Commercialization should be based upon the needs of specific markets.

The panel identified ten key issues relating to tools for design and manufacturing that must be addressed and included in the development of a common agenda:

- process modeling;
- hierarchical levels of abstraction, with seamless transition between levels;
- validation;
- requirements traceability;
- manufacturing database interoperation;
- enterprise product realization processes for workflow;
- multi-disciplinary optimization, in terms of performance and cost;
- preservation of corporate knowledge;
- design methodology and theory; and
- libraries.

### 3.3 Manufacturing Processes and Equipment

The Manufacturing Processes and Equipment panel was co-chaired by a representative from the DOE and a representative from the National Center for Manufacturing Sciences (NCMS). The panel was concerned with the issue that improved manufacturing processes and equipment are required to enable the following:

- cost effective, agile manufacturing;
- the low-cost fabrication of products made of advanced materials; and
- the exploitation of breakthroughs in processing concepts and underlying technologies, such as intelligent controls, sensors, and actuators.

In the context of this panel, manufacturing processes include both discrete and continuous manufacturing processes, and manufacturing equipment includes a broad range of computer-controlled equipment capable of carrying out a wide array of manufacturing processes. The panel focused on three main technical topics: intelligent control systems, rapid prototyping methods, and new processing methods and equipment.

Several manufacturing process and equipment issues were identified as critical to the development of a national manufacturing agenda:

- Declining R&D budgets in the private sector and lack of a short term return on investment have inhibited technical development.
- Lack of understanding of the fundamental relationships between sensed variables and required controls has delayed the integration of these technologies into manufacturing equipment.
- Affordable, robust sensors and “user friendly” controllers are not readily accessible to small, medium and large firms.
- Closer relationships are needed between U.S. equipment builders and users for early introduction of new processes and equipment into U.S. factories.

For each of the three technical focus topics, the panel made recommendations that are summarized in Table 1. The panel believes that by implementing these recommendations, a number of infrastructural benefits will be derived, such as:

- improvement in manufacturing cost, quality, throughput, and flexibility for low-to-moderate volume applications;
- facilitation of significant advances when individual technologies are integrated into advanced processing systems;
- development of new processing technologies for rapid prototyping that produce components with a broader range of mechanical properties can evolve into the “rapid fabrication” of production quality, functional parts, with a potential production of parts 10 times faster than possible by conventional manufacturing practices; and
- attainment of a significant competitive advantage in an era when time-to-market for new products is critical.

The panel concluded that these recommendations promote the realization of next generation manufacturing processes and equipment. To cultivate R&D, cooperative programs among industry, government, and academia should be established to implement development and demonstration activities. Where possible, existing partnerships and consortia should serve as the building blocks for future federally sponsored activities. These activities should then lead to the widespread implementation of new and improved manufacturing techniques.

From the perspective of the commercial machine tool industry, the panel also identified four “deadly” mistakes to be avoided in manufacturing technology implementation:

- a solution looking for a problem—“support your local professor,”
- a solution looking for a problem—“hire a representative to find the needs,”
- “pre-competitive” research—a technology R&D concern, not necessarily an implementation issue, and
- the exclusion of the commercializer.

### 3.4 Manufacturing Training and Education

The Manufacturing Training and Education panel, which focused on workforce training and education issues, was co-chaired by a representative from the NSF and a representative from the Georgia Institute of Technology.

Training and education are needed at all levels (shop floor, technical, managerial, and pre-employment) to enable businesses to make effective use of the latest production technologies. Training and education form the foundation of the manufacturing infrastructure. A well trained and educated workforce is essential for global manufacturing competition and continuous quality improvement.

The NSTC white paper on training and education identified six strategic priorities; the panel addressed these priorities.

1. Design a coherent framework.
2. Improve the quality of education and training for work.
3. Ensure access to all populations.
4. Leverage federal policy to use scarce dollars where they have the greatest impact.
5. Create supportive market mechanisms to finance manufacturing training.
6. Measure priority.

The panel made several editorial changes to the NSTC white paper, and basically concurred with most of the premises contained in it.

### 3.5 Manufacturing Deployment

The Manufacturing Deployment panel was co-chaired by a representative from the NIST MEP program and a representative from the National Association of Manufacturers. The panel focused on the premise that modern technology’s potential to enhance American manufacturing will be realized only if it is properly applied and widely used. Thus, extension programs and other technology deployment mechanisms are important elements of the nation’s manufacturing infrastructure.

In today’s business environment, the government’s role in deployment activities must be coordinated among federal agencies to support industry needs. Also, this support of industry’s needs must be demonstrated to help ensure a contribution to U.S. competitiveness.

After examining the core values, processes, and products of federal manufacturing extension programs, such as the NIST MEP, the panel made a series of recommendations regarding manufacturing deployment activities. These recommendations are summarized as follows:

- The Federal Government, with the NIST MEP playing a capacity-building and coordinating role, should work to fill the gaps in the infrastructure of technical assistance to all tiers of the industrial base.
- The Federal Government should leverage both public and private resources, serving as a catalyst for action.
- The Federal Government should avoid duplicating functions offered elsewhere in the manufacturing deployment services market.
- The federal role, based upon its national perspective, should include functions such as information dissemination, performance metrics and standards, best practices, electronic linkages, lessons learned, and benchmarking information.
- The Federal Government must provide the mechanism for incorporating the needs of small-to-medium size enterprises in the national research agenda.
- As competent service providers and small manufacturers overcome barriers to change, such as lack of information, isolation, regulatory burdens, and financing, the federal role must evolve. This evolution will occur as the market and economy absorb technical assistance functions

In the definition of the federal role in manufacturing deployment activities, two issues are critical. First, the ultimate customer of deployment services is the U.S. manufacturing base. Second, the immediate customer is the extension network of service providers.

### 3.6 Business Practices

This panel was co-chaired by a representative from the Advanced Research Projects Agency (ARPA) and a representative from Computer Aided Manufacturing-International (CAM-I). The focus of the panel was on practices that permit companies to create flexible and readily-adaptable enterprises. Such practices are rapidly changing the ways in which companies do business. These practices, such as “lean” and “agile” manufacturing, are changing the ways in which companies assemble production resources, interact with their suppliers, and respond to changing customer needs.

In addressing business practice issues, the panel identified the key characteristics of the next generation of manufacturing systems that will be enabled by changes in business practices. The following attributes of these next generation manufacturing systems were identified:

- customer-focused, business-oriented;
- reconfigurable, adaptable, flexible;
- modular to support distribution and autonomy;
- support for global design and production;
- rich in human intelligence;
- cooperative to support enterprise goals;
- support for the virtual enterprise;
- information- and knowledge-based; and
- environmentally aware.

When addressing improved business practices in manufacturing, the panel identified three primary areas of interest for the government:

- economic growth;
- fairness and other social goals; and
- affordability and stewardship, where the government is the customer.

The panel agreed that government’s behavior as a customer has a strong influence on the nature of business practices. The panel also determined that addressing commercial business practices is essential to the other five NSTC manufacturing infrastructure areas of emphasis. As such, the panel recommended that Business Practices be added as the sixth area of emphasis.

## 4. Summary

The second annual “Manufacturing Technology Conference: Toward a Common Agenda,” held at NIST in April 1995, provided a 3 day broad-based national forum to define a national manufacturing agenda. Manufacturing technology issues were presented and discussed by representatives from government, industry, and academia on policy, program, and technology levels.

In addition to serving as a vehicle for information exchange, the Conference made significant progress in defining the critical elements of the nation’s manufacturing infrastructure. This national manufacturing infrastructure is being created in conjunction with the President’s NSTC. The goal is the establishment of a common manufacturing agenda for the nation.

A common manufacturing agenda, which involves all sectors of the nation, will lead to the increased ability of U.S. manufacturers of all sizes and industries to compete in the global economy.

## Figures and Tables

**Table 1:** Recommendations of the Manufacturing Processes and Equipment Panel

Technical focus	Near-term (1–3 years)	Mid-term (3–5 years)	Long-term (5–10 years)
Intelligent control systems	• sensors, models, and knowledge-based algorithms • cooperative national effort to characterize parameters in a standard format • develop verification methodology for process models	• expand process models to include additional manufacturing processes and “non-traditional” processes • develop and deploy standard open-architecture controllers	• develop neural networks intelligent manufacturing equipment • develop and demonstrate knowledge-based artificial intelligence manufacturing systems in small, medium, and large companies in several sectors
Rapid prototyping	• improve part accuracy and fabrication speed • introduce new materials and processes to create functional parts	• demonstrate systems developed in the near term • demonstrate rapid prototyping technologies for new materials	• develop “rapid fabrication” of production quality functional parts made on similar time scale as by rapid prototyping
New processing methods and equipment	• develop a process to reduce the cost of advanced materials for structural applications by a factor of 10 • develop flexible and modular tooling and equipment adaptable to a variety of sensors and controls	• develop and demonstrate a reconfigurable manufacturing system with modular hardware, tooling, sensors, and controls • develop control system for retrofits on existing manufacturing equipment	• develop processes to red the cost of advanced materials for structural applications by a factor of 100 • develop and demonstrate precision manufacturing systems that can be reconfigured for low and high volume processes
